# Age-Friendly Communities and Older Adults’ Health in the United States

**DOI:** 10.3390/ijerph19159292

**Published:** 2022-07-29

**Authors:** Kyeongmo Kim, Thomas D. Buckley, Denise Burnette, Jin Huang, Seon Kim

**Affiliations:** 1 School of Social Work, Virginia Commonwealth University, Richmond, VA 23284, USA; jdburnette@vcu.edu (D.B.); kims@vcu.edu (S.K.); 2 Department of Psychiatry, School of Social Work, University of Pittsburgh, Pittsburgh, PA 15260, USA; tdb47@pitt.edu; 3 School of Social Work, Saint Louis University, St. Louis, MO 63103, USA; jin.huang@slu.edu

**Keywords:** age-friendly environments, age-friendly community, health, well-being, livability, physical environment, social environment

## Abstract

As age-friendly community (AFC) initiatives grow, it will be essential to determine whether older adults who live in an AFC have better health than those who live in other environments. This study uses data from the 2017 AARP AFC Surveys and the AARP Livability Index to assess whether AFCs promote the health of older adults. We analyze data for 3027 adults aged 65 and older who reside in 262 zip code areas. Following AARP guidelines, we allocated the sample into two groups: an AFC group (livability score of 51+; *n* = 2364) and a non-AFC (score ≤ 50, *n* = 663). The outcome variable was self-rated health (M = 3.5; SD = 1.1; range: 1–5). We used an inverse probability weighting approach to evaluate whether older adults who live in an AFC reported better self-rated health than those who live in a non-AFC. Findings showed that older adults who lived in an AFC had better self-rated health than those in a non-AFC (b = 0.08, *p* = 0.027). Compared to non-Hispanic Whites, Black and Hispanic older adults reported worse self-rated health. Inasmuch as living in an AFC can promote the well-being of older adults, policymakers and practitioners should continue to develop and sustain high-quality, accessible built and social environments.

## 1. Introduction

The World Health Organization (WHO) Age-Friendly Cities (AFC) initiative was designed to promote active, healthy aging and to help cities plan for rapid population aging [1,2]. Beginning in 2007, the WHO AFC initiative originally included a global network of 33 cities and communities in 22 countries, and identified key characteristics necessary for developing an age-friendly city. In 2010, the WHO formally introduced the Global Network of Age-friendly Cities and Communities (GNAFCC) to promote the collaborative exchange of ideas, resources, and best practices [3]. This global network has since expanded to include over 298 million people in 1333 cities and communities in 47 countries [4]. These AFCs implement policies and programs that aim to improve older adults’ communities and neighborhoods, increase healthy life spans, and enhance the quality of life [5]. To improve the fit between older adults’ needs and their physical and social environments, they focus on eight key interrelated domains: outdoor spaces and buildings, transportation, housing, social participation, respect and social inclusion, civic participation and employment, communication and information, and community support and health services [2].

Most studies link AFC features to superior outcomes. For example, among national samples of community-dwelling older adults in the US, individuals who rate their community as more “age-friendly” reported better health [6,7]. This same relationship was found in samples of older adults in urban areas in the US [8] and Hong Kong [9]. Regarding mental health, AFCs are linked to lower depressive symptoms [10] and better mental health-related quality of life [11]. Other studies found AFC characteristics are related to better life satisfaction in urban areas in Hong Kong and China [12,13], as well as better quality of life among older adults in urban areas in Ireland [10]. Last, a recent scoping review found that the built and social environments of AFC are related to positive health and well-being outcomes, and that this relationship is often mediated by a psychological sense of community [14].

However, strong empirical evidence of the effects of these communities on health outcomes is still limited and needs to be further evaluated [15,16]. As AFCs grow, it will be important to determine whether they confer a health advantage for older adults. There is a dearth of research comparing those who live in age-friendly environments with those who do not. The current study joins growing efforts to answer that question by addressing the potential adverse selection bias effects of AFC evaluations (e.g., [7]). Older adults may self-select themselves into a neighborhood that meets their needs [7]. For example, individuals may consider their health when they choose a neighborhood that they want to live in [17]. Further, there is a need to use valid and reliable measures for comparing different communities [18]. The current study aims to fill these gaps by using a nationwide measure of age-friendliness developed by the AARP [19] to explore whether residing in an age-friendly community is associated with better health.

### 1.1. Conceptual Framework

The rapid and divergent growth of AFC programs has outpaced theoretical accounts of their developmental processes and outcomes [20,21]. In addition, programs focus on different domains and lack a valid, uniform measure of ‘age-friendliness,’ which inhibits rigorous evaluations of their effects within and across communities. For example, although programs ideally assign equal weight to physical and social environments, most emphasize the former. These despite the fact that social interventions are often more readily accomplished and evaluated than large infrastructure measures [22].

For nearly a half-century, Lawton and Nehemow’s [23] Ecological Theory of Aging (ETA) has served as a basis for conceptualizing the aging process. Owing to its focus on the continual interaction of older adults’ capabilities and functioning with their physical and social environments, the ETA has emerged as a key organizing framework for the AFC movement [24]. This framework explores the goodness-of-fit between an older adult and their environment, whereby a good person-environment (P-E) fit leads to positive outcomes and a poor P-E fit to negative outcomes. AFC environments are meant to improve health and well-being outcomes by proactively addressing P-E fit.

Similarly, livability theory addresses the extent to which a living environment fits the adaptive repertoire of a species. In human societies, it refers to the fit of institutional arrangements with human needs and capacities [25]. Applying livability theory to older adults, AARP [26] defines a livable community as one that has “affordable and appropriate housing, supportive community features and services, and adequate mobility options, which together facilitate personal independence and the engagement of residents in civic and social life.” The World Health Organization’s eight interconnected domains that characterize livable cities can help to identify and address barriers to older adults’ well-being and social participation. We use ETA and livability theory as a framework to understand *why* AFC may improve the health of older adults.

In addition to shortcomings related to theoretical development, most studies rely on subjective perceptions of age-friendliness, although objective data are also needed [27]. Zhang et al. [28] found that per capita public space and service density of neighborhood senior services were associated with better well-being. Using a mix of subjective and objective measures of the built environment, Guo et al. [29] reported that incorporating the latter, e.g., amount of green space and service availability and accessibility, added to understanding mental health and well-being outcomes. Finally, in comparing subjective perceptions of community residents and objective assessments of policymakers and government officials, Menec et al. [27] found that these groups had congruent understandings of age-friendliness in their community, which suggests a potential avenue for objective evaluation of age-friendliness.

### 1.2. AARP Livability Index

The AARP [19] Livability Index is a publicly available objective measure of the ”age-friendliness” of local communities in the US The instrument defines livability in terms of community characteristics that support independence, choice, and safety, and foster the ability of older adults to thrive in their environment. The Livability Index provides ratings for important dimensions of one’s community: housing, neighborhood, transportation, environment, health, engagement, and opportunity. Ratings are determined from data collected from local, state, and federal government as well as private sources. Data types range from policies and service availability to census and health monitoring data. Specific scoring metrics for each dimension are provided by the AARP [30]. Scores for each dimension are averaged for an overall livability score, which is calculated either by ZIP codes or by cities/counties. The Livability Index score, in theory, ranges from 0 to 100; a score of 50 refers to the national average. In general, higher ratings equate to a more age-friendly and livable environment. This index represents a uniform measure that can be used to compare different communities. The Livability Index is a potentially important resource for older adults and their families in evaluating their current or prospective living environments and for policymakers in identifying intervention targets [31].

More researchers are also now using the Livability Index to, for example, compare health equity in rural and urban areas [32], assess its relationship with mobility and migration in rural communities [33], and as a control variable in empirical analyses [34]. Research has yet to examine the Livability Index with health outcomes. However, the development of the index is grounded in the WHO AFC framework [2] and P–E fit, both of which propose that age-friendly environments should lead to better health outcomes for older adults. The current study advances knowledge on the role of AFCs in older adults’ health by combining zip code level Livability Index scores with data from AARP AFC surveys. Based on previous findings, we hypothesized that older adults who live in AFCs will have better health than those who live in non-AFCs. We tested this hypothesis using the inverse probability weighting approach to adjust for self-selection bias.

## 2. Methods

### 2.1. Data Source and Sample

Data are from the 2017 AARP Livability Index and the 2017 AARP AFC Surveys. The AFC Surveys are designed to identify the needs of older adults in order to promote their ability to age in place [35]. AARP research teams selected 13 US metropolitan areas and 1 community in Puerto Rico on the basis of geographic locale and demographic composition: Albany County, NY; Bernalillo County, NM; Davidson County, TN; Greater East Lansing, MI; Greater Kanawha Valley, WV; Greater Rapid City, SD; Miami-Dade County, FL; Milwaukee, WI; San Juan, PR; Seattle, WA; Springfield, IL; Tucson, AZ; Warwick, RI; Wilmington, NC. In these communities, Alan Newman Research conducted a population research study using landline and cell phone samples (random digital dial survey). They conducted 30 min telephone interviews with adults aged 45 and older between June 2017 and September 2017 (N = 6670). Using the methodology of the American Association for Public Opinion Research, the team reported three response rates: average participation (49.1%), refusals (11.3%), and non-responses (4.4%). AARP [36] provides detailed information on its survey methodologies by area.

We obtained Livability Index data from the AARP Public Policy Institute and merged Index scores with AFC survey data by zip code. Our sample is restricted to respondents who: (a) were aged 65 and older, (b) lived in the USA, and (c) had a valid Livability score. Out of 6670 survey respondents, we excluded people who lived in Puerto Rico (*n* = 504), were aged below 65 (*n* = 2929), and lived in an area without a livability score (*n* = 25). Missingness of included variables ranged from zero to three percent and we excluded respondents with missing values (*n* = 185) after confirming missing at random by Little’s MCAR test that was not significant. Our analyses are based on 3027 adults aged 65 and older living in 262 zip codes.

### 2.2. Measures

#### 2.2.1. Age-Friendly Community

We measured the age-friendliness of a community using a Livability Index score ranging from 0 to 100. AARP created the scores by comparing the age-friendliness of communities. For example, they conceptualized a livability score of 50 as the average community. A score of 51 and over refers to above-average communities, while a score of 50 and below indicates average or below-average communities [36]. Following AARP guidelines, we allocated the sample into two groups: AFC (livability score of 51+, *n* = 2364) and non-AFC (score ≤ 50, *n* = 663).

#### 2.2.2. Self-Rated Health

We assessed self-rated health using the single-item self-report measure: “In general, would you say your health is…” (1 = poor, 2 = fair, 3 = good, 4 = very good, 5 = excellent). Higher scores indicate better self-rated health (M = 3.44, SD = 1.11, range: 1–5). We treated the measure as continuous (skewness = −0.38).

#### 2.2.3. Covariates

We adjusted for demographic and health-related characteristics associated with neighborhood selection and physical health. Demographic characteristics included age (1 = 65–74, 2 = 75–84, 3 = 85 and older); sex (0 = male, 1 = female); education (1 = 0 to 12th grade, but with no diploma, 2 = high school graduate or equivalent, 3 = post-high school education, but with no degree, 4 = two year degree like an associate’s or vocational degree, 5 = four year degree such as bachelor’s degree 6 = postgraduate study, but with no degree, 7 = graduate or professional degree); currently employed (0 = no, 1 = yes); homeownership (0 = no, 1 = yes); at least five years of residence in current community (0 = no, 1 = yes); and living arrangement (0 = living alone, 1 = living with spouse or partner). Health-related questions asked whether respondents have physical limitations (0 = no, 1 = yes) and whether they are current family caregivers (0 = no, 1 = yes). Finally, we included a social interaction measure, i.e., frequency of contact with family, friends, or neighbors who do not live with respondents (0 = never, 1 = less than monthly, 2 = once a month, 3 = once every two or three weeks, 4 = once a week, 5 = several times a week, 6 = every day).

### 2.3. Data Analysis

To evaluate whether older adults who live in an AFC reported better self-rated health than those in a non-AFC, we used linear regression with inverse probability weights (IPW) [37,38]. As it is not possible to randomly assign residents to the treatment group (i.e., AFC) or control group (i.e., non-AFC), we could not determine whether a finding of better self-rated health status was attributable to age-friendliness or existing characteristics of respondents who live in the community. Thus, the IPW helps us make the “treatment” and “control” groups comparable to reduce the selection bias inherent in observational studies [37,38].

First, we estimated the probability of group membership using individual characteristics associated with neighborhood selection (i.e., age, sex, education, employment, homeownership, living arrangement, social interaction, physical limitation, and caregiving experience). After ensuring common support from the treatment and control group, we created the propensity weight for the average treatment effect on the treated (ATT). Next, we compared the two groups’ unweighted means and weighted means to determine whether the groups were equivalent. After checking group differences, we employed linear regression using the ATT weighting variable. We used pairwise deletion because missing data were below two percent, with the exception of race/ethnicity (3%). We used a one-tailed test as we hypothesized that living in an AFC would lead to better health.

## 3. Results

Table 1 presents selected sample characteristics. Over half of the respondents were aged 65 to 74, and 59% were female. The majority were non-Hispanic White (83.6%), followed by Hispanic (7.8%), Black (6.6%), Other (i.e., Asian, Native American, Alaskan Native, Native Hawaiian, Pacific Islander, or some other) (2.1%). Over 90% of respondents had lived in their current community for at least five years, and they maintained high levels of social interaction with family and/or friends. The sample characteristics differed by where the respondents lived. Older Whites were more likely to live in AFCs, whereas Black and Hispanic older adults lived in non-AFCs. Older adults with higher levels of education lived in AFC.

Table 2 presents descriptive statistics for covariates before and after ATT weighting and information on the equivalency of weights for the treatment and control groups. A standardized mean difference below 0.1 indicates a negligible imbalance between the AFC and the non-AFC group (see [39]). We found differences in means in the unweighted sample (e.g., ages 85 and older and race/ethnicity). After applying the weights, the standardized mean differences were less than 0.05. The covariates did not differ significantly for the weighted sample and thus required no statistical adjustment.

Table 3 presents multivariate regression models for self-rated health. Older adults living in an age-friendly community had better self-rated health than those living in a non-age-friendly community, adjusting for all other covariates (b = 0.08, *p* = 0.029). Individuals aged 85 and older had better self-rated health than those aged 65–74. Hispanic and Black respondents had significantly lower self-rated health than their White counterparts (b = −0.23, *p* = 0.001; b = −21, *p*= 0.006, respectively). Respondents with higher education, employment, and home ownership reported significantly better self-rated health. As expected, those who reported more frequent social interaction had better self-rated health and those with physical limitations had worse self-rated health. We also conducted an ordinary least square analysis using the livability score as continuous and the findings were consistent with the results presented in Table 3 (data not shown).

## 4. Discussion

Studies of AFCs suggest that creating physical and social environments favorable to the needs of older adults can promote better health; however, since older adults may in theory choose their neighborhoods to support their daily living, adverse selection bias may affect the evaluation of these programs (see [40]). We thus used the inverse probability weight approach to adjust for neighborhood self-selection bias.

There is abundant evidence that health is socially determined across the life course and that the quality of the built environment significantly affects health. As Browne and Lowe [41] point out, the domains of the Livability Index, i.e., the quality and quantity of public open space, active transportation venues, access to healthy food and other goods, and opportunities for recreation, employment, and education, all show causal links to good health. For planning and evaluation purposes, it is important to determine the mechanisms that account for these links in different populations. Xu et al. [42] found that a higher level of age-friendliness in a community is associated with lower odds of frailty, for example. Services to detect and treat mental health problems, which may directly and indirectly affect health and physical activity, cognitive function, and premature mortality are also important areas of prevention and intervention [43]. Finally, examination of physiological and psychosocial dynamics of the aging process, including similarities and differences in young-old and older age groups, is a significant line of research on age-friendly communities [44].

Results of our analyses before and after applying the IPW support our hypothesis that after adjusting for relevant covariates and self-selection into the neighborhood, AFC environments confer health advantages for older adults. Consistent with the previous finding that older Whites were more likely to live in more livable communities than people of color [45], they lived in an AFC in this study. As education is a consistent factor to choose a neighborhood [46], those with higher levels of education were more likely to live in an AFC. As noted, these communities are highly variable, with multidimensional, interdependent features [16]. For example, easy access to the neighborhood environment (e.g., park or senior center) can provide more opportunities for physical activities and social interactions [40]. Likewise, the mode of transportation can affect one’s ability to participate in health-promoting and social activities [47,48]. Multi-sector collaborations are thus essential to improving both the quality and reach of age-friendly environments [49,50].

To guide such collaborations, future research should identify components of the complex concept of age-friendliness while striving for consensus on basic AFC measures. The WHO concept of “intrinsic capacity” may be useful for assessing and tracking change in individual functioning. Defined as a composite measure of a person’s physical and mental capacities, it is conceptualized as a dynamic construct with five domains (vitality, locomotor, sensory, cognitive, and psychological) [51]. Likewise, resources exist and interact in different ways in different communities. For example, transit-rich neighborhoods may not score high on housing because housing prices may rise [36].

Identifying the dynamics of intrinsic capacities and their fit with certain complements of resources and services in a given environment will enable more effective and efficient targeting of services. Longitudinal data are needed to disentangle issues of temporality and causality and to improve understanding of the shifting qualities and dynamics of “fit” between older adults’ interactions with their physical and social environments. Peek et al. [52] suggest the use of a dynamical systems theory approach, including technology, for this purpose. As Menec [27] notes, the notion of fit requires holistic, interdisciplinary research since age-friendly domains cannot be treated in isolation from intrapersonal factors.

Improving assessment, evaluation, and outcomes also requires policymakers, practitioners, and researchers to ensure the meaningful, sustained involvement of community stakeholders, including more vulnerable subpopulations of older adults. The WHO [53] cites equity, physical accessibility, and social inclusiveness as key principles in evaluation, while Meeks [16] cites inclusiveness as the biggest challenge. Indeed, since socially and economically vulnerable people are more likely to live in poor neighborhoods, the AFC model can inadvertently exacerbate health inequities [54].

Most AFC initiatives mention targeted efforts to include diverse subgroups of older adults, yet their involvement is only moderate [15]. In the current study, for example, Black and Hispanic older adults had worse health, and AFCs had a greater impact on the health of the oldest-old. There are several possible reasons for our finding that individuals aged 85 and older had better self-rated health than those aged 65–74. The first explanation concerns survival bias, as the population of adults aged 85 and older who live in the community has not been censored by death or the need for more intensive care environments, and they may thus be a healthier group than the young-old. Second, self-rated health is a mix of biology, psychological state, and culture [55]. As people age, they lower their standards of what constitutes good health and adjust their ratings accordingly [56]. Self-ratings among the oldest-old may thus not be the same as those of younger old adults. Finally, self-rated health may be affected by the context in which the question is asked. For example, in a large US survey, Bozick [57] assessed the validity of self-rated health in predicting the risk of mortality by comparing respondents who rated their health before and after reporting their body weight. The odds of more favorable health appraisals were 30% lower for the latter group. Ratings of self-rated health may thus vary by demographic factors, including age, depending on the salience and context of the question for a given age group. Future research should aim to disentangle these and other possible reasons for differences in self-rated health by age group within the older adult population.

Future studies should also involve older adults from a full range of racial and ethnic backgrounds [58], age strata, low-income individuals and communities at higher risk of negative health outcomes [59], frail older adults [60], rural communities [61], and international and migratory populations [62].

Greater attention is also needed to cultural aspects of health beliefs, attitudes, and behaviors and to outreach and implementation strategies that maximize service access and uptake. Healthy older adults who live in AFCs may play a key role in such efforts. Qualitative research can be especially useful for deepening knowledge and building theory about barriers and facilitators to active involvement in monitoring changes in themselves and their social and physical environments [60].

As the Livability Index is derived from census data, policies, and neighborhood programs, it provides a more comprehensive measure of age-friendliness. As with other objective measures, e.g., public officers’ views [27], age-friendliness of the built environment [29], and availability of services and public space [28], the Index is a valuable complement to subjective assessments in seeking a fuller understanding of AFCs and facilitating comparisons across sites and over time.

This study has several limitations. As our sample was from 13 US metropolitan areas, and most respondents were non-Hispanic White, we cannot generalize our findings to older adults throughout the country or those from other demographic backgrounds. As the number and proportion of racial and ethnic minority older adults increase, the needs of subpopulations will become more diverse, and existing disparities in accessing services could worsen. In our study, Black and Hispanic older adults reported worse health status than Whites. Again, future research should determine whether, to what extent, and in what ways, older adults from diverse racial and ethnic groups benefit from AFC initiatives. Another possible limitation is the use of the subjective measure of self-rated health, which can be less reliable and more contextual than objectively measured health status [63,64]. However, self-rated health is a valid measure of overall health status [64,65], can be effectively used to identify vulnerable older adults’ health status with a single item [66], and is widely used in national health surveys [6].

The AARP AFC data are from a cross-sectional study design, which limits our ability to infer a causal relationship between age-friendly environments and health outcomes. Although we established equivalent treatment and control groups using IPW, there may be unobservable sample characteristics associated with neighborhood selection. As noted, since older adults and their neighborhood environments interact and change over time, longitudinal designs are needed to understand how living in an AFC might affect individuals’ health. Despite these limitations, this study is the first to evaluate the effect of AFC initiatives on health outcomes of older adults using objective measures of age-friendliness (i.e., AARP Livability Index) and individual health outcomes (i.e., self-rated health) from AFC Surveys while adjusting for potential selection bias.

## 5. Conclusions

The current study suggests that older adults who live in an AFC have better health status than those who live in non-AFCs. Where people live can be influenced by their personal characteristics (e.g., race/ethnicity and socioeconomic status) and questions remain about who lives in age-friendly neighborhoods. This study extends the existing literature by addressing self-selection issues in neighborhoods and adds to the growing literature on the role of age-friendly environments in older adults’ health, and suggests directions for future research. As adults age, the built and social environments become more critical. Since AFCs can promote their well-being, policymakers and practitioners should continue to build high-quality, accessible built and social environments.

## Figures and Tables

**Table 1:** Sample characteristics by age-friendliness.

Variables	M (SD)/N (%)	AFC (*n* = 2364)	Non-AFC (*n* = 663)
Age			
65–74	1752 (57.88)	57.66	58.67
75–84	837 (27.65)	27.16	29.41
85 older	438 (14.47)	15.19	11.92
Sex at birth (Female)	1795 (59.30)	59.73	57.77
Race/ethnicity ***			
White, non-Hispanic	2529 (83.55)	85.58	76.32
Black, non-Hispanic	199 (6.57)	5.71	9.65
Hispanic	235 (7.76)	6.56	12.07
Other, non-Hispanic	64 (2.11)	2.16	1.96
5 years of residence in the community (yes) *	2818 (93.10)	93.70	90.95
Education *			
0 to 12th grade, but with no diploma	127 (4.20)	3.81	5.58
High school graduate or equivalent	588 (19.43)	19.71	18.40
Post-high school education, no degree	440 (14.54)	13.66	17.65
2-year degree	418 (13.81)	13.62	14.48
4-year degree	634 (20.94)	21.36	19.46
Postgraduate study, no degree	170 (5.62)	5.92	4.52
Graduate or professional degree	650 (21.47)	21.91	19.91
Currently employed (yes)	514 (16.98)	16.96	17.04
Owns home (yes)	2519 (83.22)	83.84	81.00
Living with spouse/partner (yes)	1518 (50.15)	50.08	50.38
Social interaction (0–6) (Mean) *	5.1 (1.3)	5.1 (1.2)	5.0 (1.3)
Physical limitation (yes)	896 (29.60)	29.19	31.07
Current caregiver (yes)	460 (15.20)	15.36	14.63

* *p* < 0.05, *** *p* < 0.001.

**Table 2:** Covariate descriptive before and after ATT weighting.

Covariates	Unweighted Sample			Weighted Sample		
	AFC	Non-AFC	Std. Difference	AFC	Non-AFC	Std. Difference
Age						
65–74	0.58	0.59	−0.02	0.58	0.57	0.02
75–84	0.27	0.29	−0.05	0.27	0.27	0
85 and older	0.15	0.12	0.1	0.15	0.16	−0.03
Sex (Female)	0.6	0.58	0.04	0.6	0.6	−0.00
Race/ethnicity						
White	0.86	0.76	0.24	0.86	0.86	−0.00
Black	0.06	0.1	−0.15	0.06	0.06	−0.01
Hispanic	0.07	0.12	−0.19	0.07	0.07	0
Other	0.02	0.02	0.01	0.02	0.02	0.01
Years in community	0.94	0.91	0.1	0.94	0.94	−0.00
Education	4.34	4.17	0.09	4.34	4.31	0.02
Employment	0.17	0.17	−0.00	0.17	0.17	−0.01
Own Home	0.84	0.81	0.08	0.84	0.83	0.02
Living with spouse/partner	0.5	0.5	−0.01	0.5	0.5	0.01
Social interaction	5.1	4.99	0.09	5.1	5.09	0.01
Physical limitation	0.29	0.31	−0.04	0.29	0.3	−0.02
Caregiving	0.15	0.15	0.02	0.15	0.15	0

Note. AFC = age-friendly community; Std. difference = standardized mean difference.

**Table 3:** Linear regression of age-friendliness on self-rated health with inverse probability weight.

Variable	Coef.	Robust SE	95% CI	
			Low	High
Age-friendly community	0.08 *	0.04	−0.00	0.17
Age				
65 and older (ref)	-	-	-	-
75–84	−0.04	0.05	−0.14	0.07
85 and older	0.27 ***	0.07	0.13	0.41
Sex (Female)	0.03	0.05	−0.07	0.12
Race/ethnicity				
White (ref)	-	-	-	-
Black	−0.23 **	0.07	−0.38	−0.09
Hispanic	−0.21 **	0.07	−0.35	−0.05
Other	−0.12	0.16	−0.42	0.19
5 years in Community (yes)	−0.09	0.08	−0.25	0.06
Education	0.08 ***	0.01	0.05	0.1
Employment (yes)	0.25 ***	0.06	0.12	0.38
Own home (yes)	0.20 **	0.06	0.08	0.33
Living with spouse/partner	−0.04	0.05	−0.13	0.05
Social interaction	0.06 **	0.02	0.02	0.09
Physical limitation (yes)	−1.01 ***	0.05	−1.10	−0.90
Caregiver (yes)	0.08	0.06	−0.04	0.2
Intercept	3.21 ***	0.15	2.93	3.5

Note. Coef = coefficient; SE = standard error; CI = confidence interval. * *p* < 0.05, ** *p* < 0.01, *** *p* < 0.001.

## Data Availability

Not applicable.
